# Non-Linear Enthalpy-Entropy Correlation for Nitrogen Adsorption in Zeolites

**DOI:** 10.3390/molecules23112978

**Published:** 2018-11-15

**Authors:** Montserrat R. Delgado, Carlos O. Arean

**Affiliations:** Department of Chemistry, University of the Balearic Islands, E-07122 Palma, Spain; montserrat.rodriguez@uib.es

**Keywords:** enthalpy-entropy correlation, IR spectroscopy, nitrogen adsorption, zeolites

## Abstract

The thermodynamics of dinitrogen adsorption in faujasite-type zeolites, Na-Y, Ca-Y and Sr-Y, were investigated by means of variable-temperature infrared spectroscopy, a technique that affords determination of the standard adsorption enthalpy (Δ*H*^0^) and entropy (Δ*S*^0^) from an analysis of the IR spectra recorded over a range of temperatures. The results obtained, taken together with previously reported values for N_2_ adsorption on protonic zeolites, revealed a non-linear correlation between Δ*H*^0^ and Δ*S*^0^. Implications of such a correlation for gas separation and purification by adsorption in porous solids are highlighted.

## 1. Introduction

By virtue of their (relatively) high thermal stability, large gas adsorption capacity, and low cost, zeolites (and related porous solids) are currently used in industrial processes, such as oxygen and nitrogen production from air, upgrading (sweetening) of natural gas, and purification of hydrogen obtained from steam reforming of hydrocarbons or from syngas [1,2,3,4,5], to quote only a few examples. Future applications of zeolites as selective gas adsorbents can also be envisaged, such as in carbon capture and sequestration (CCS) from the flue gas of coal-fuelled power stations and for improving indoor air quality inside submarines and manned spacecraft [6,7,8,9,10,11].

In addition to their stability and low cost, an intrinsic advantage of zeolites over other gas adsorbents, such as activated carbons and porous polymers [12,13], is the facility with which cation exchange can be carried out on zeolites, which provides a means to tune the strength of the electrostatic field at the cation sites that constitutes a major factor determining gas-solid interaction energy. It should be noted, however, that thermodynamic (equilibrium) gas separation, storage and delivery are ruled by both adsorption enthalpy (Δ*H*^0^) and entropy (Δ*S*^0^), which together determine the Gibbs free energy of the process. Moreover, an enthalpy-entropy correlation, frequently referred to as compensation [14,15], could be non-linear, as found some time ago in the case of hydrogen adsorption in zeolites [16,17]. Herein we give corresponding values for nitrogen, as measured by variable-temperature IR (VTIR) spectroscopy. An abridged outline of this experimental method is given below, to facilitate understanding for non-specialized readers.

## 2. Outline of the VTIR Method

Variable-temperature infrared (VTIR) spectroscopy is an instrumental technique [18,19] that allows one to obtain, not only the spectroscopic signature of a gas-solid adsorption complex, but also the magnitude of the standard enthalpy (Δ*H*^0^) and entropy (Δ*S*^0^) involved in the adsorption process, provided that adsorption brings about a change in a characteristic IR absorption mode of the gas molecule, or gives origin to such a vibration mode. Whenever that is the case, let Equation (1) below describe the gas adsorption equilibrium:

*S*_(s)_*+ M*_(g)_ ⇆ *S-M*_(ads)_ (1)

For an ideal system, the integrated intensity of the characteristic IR absorption band being monitored should be proportional to surface coverage, θ, of the adsorption sites. Hence, that intensity (absorbance) gives information of the activity (in the thermodynamic sense) of both the adsorbed species and the empty sites (1 − θ). Simultaneously, the gas equilibrium pressure, *p*, gives the activity of the gas phase. Therefore, by measuring IR absorbance and equilibrium pressure at any given temperature, the equilibrium constant, *K*, at that temperature can be determined; and the variation of *K* with *T* (along a series of IR spectroscopic measurements taken over a temperature range) is related to Δ*H*^0^ and Δ*S*^0^ through the well-known van’t Hoff equation:

*K*(*T*) = exp(−Δ*H*^0^/*RT*) exp(Δ*S*^0^/*R*) (2)

Assuming Langmuir-type adsorption, combination of Equation (2) with Equation (3) leads to Equation (4) below:

θ = *K*(*T*)*p*/[1 + *K*(*T*)*p*] (3)

ln {θ/[(1 − θ)*p*]} = (−Δ*H*^0^/*RT*) + (Δ*S*^0^/*R*) (4)

Equation (4) can also be written as:

ln {*A*/[(*A*_M_ − *A*)*p*]} = (−Δ*H*^0^/*RT*) + (Δ*S*^0^/*R*) (5)

where *A* stands for the actual IR intensity being measured and *A*_M_ is the maximum absorbance at full surface coverage (θ = 1). It is thus clear that, after recording IR absorption and equilibrium pressure over a temperature range, a van’t Hoff plot of Equation (4) or (5) gives direct access to the corresponding values of Δ*H*^0^ and Δ*S*^0^ which characterize the thermodynamics of the gas-solid adsorption process. Details of the assumptions made, and hence on the applicability of Equations (4) and (5) can be found elsewhere [19,20].

## 3. Experimental Protocol

The Na-Y, Ca-Y and Sr-Y zeolites used herein were obtained by repeated ion exchange of portions of the same parent NH_4_-Y sample (Zeolyst, Si:Al = 2.55) with a 0.5 M solution of the corresponding (Na, Ca and Sr) nitrate; total ion exchange was checked by IR spectroscopy. Powder X-ray diffraction showed (in all cases) good crystallinity and absence of any diffraction line not corresponding to the FAU structure type.

**Figure 1:**
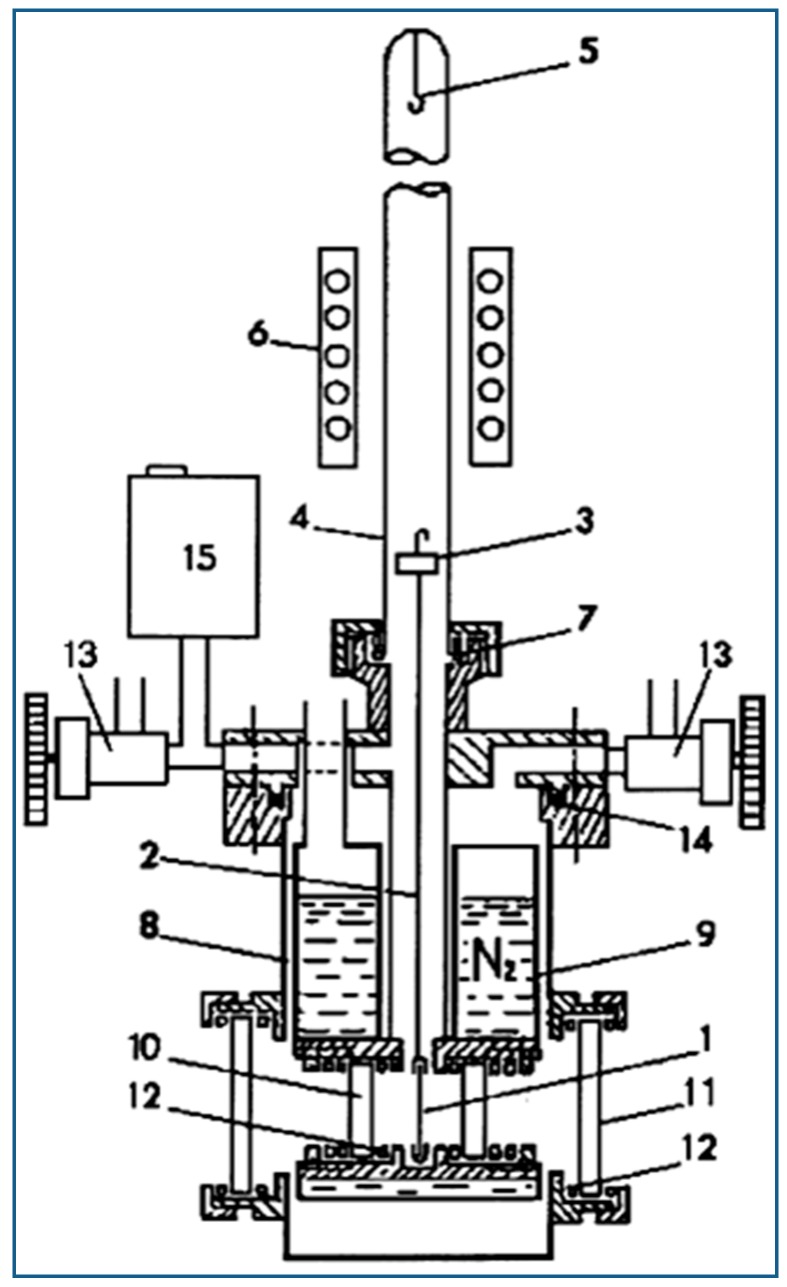
Scheme of the homemade (stainless-steel) variable-temperature IR cell: (1) sample wafer, (2) sample holder, (3) magnetically driven anchoring piece, (4) quartz tube, (5) hook for fixing the sample wafer inside the furnace, (6) furnace, (7) Viton O-ring, (8) cell body, (9) refrigerated region, (10) and (11) optical windows, (12) indium gaskets, (13) valve, (14) Teflon gasket, (15) pressure gauge. Refs. [21,22].

For VTIR spectroscopic measurements, thin self-supported wafers of the zeolite samples were prepared and activated (outgassed) in a dynamic vacuum (residual pressure smaller than 10 ^−4^ mbar) for 5 h at 650 K inside an IR cell [21,22] (shown in the Figure 1) that allowed in situ sample activation, gas dosage and variable-temperature IR spectroscopy to be carried out. After sample activation, the cell was cooled with liquid nitrogen and dosed with an amount of nitrogen gas small enough to prevent formation of geminal M^n+^(N_2_)_2_ adsorbed species (M = Na, Ca, Sr), which would otherwise complicate determination of the corresponding IR absorbance [23]. The cell was then closed, and series of VTIR spectra were recorded (upon gradual warming up of the IR cell) while simultaneously registering temperature and gas equilibrium pressure. For this purpose, the cell was equipped with a platinum resistance thermometer (Tinsley) and a capacitance pressure gauge (MKS, Baratron). Transmission FT-IR spectra were recorded, at 2 cm^−1^ resolution, using a Bruker Vertex 80v spectrometer. Sixty-four scans were accumulated for each spectrum.

## 4. Results and Discussion

### 4.1. Dinitrogen Adsorption in Na-Y

**Figure 2:**
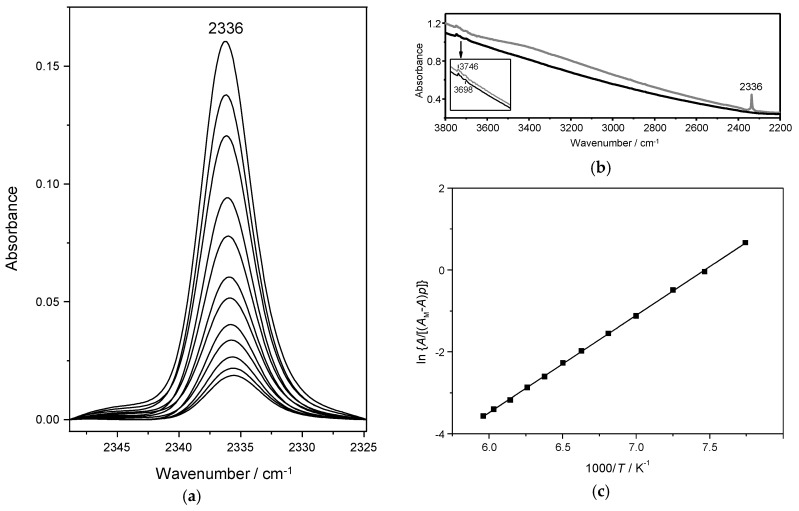
(**a**) Variable-temperature IR spectra (in the N–N stretching region) of N_2_ adsorbed in Na-Y. From top to bottom temperature increases from 129 to 177 K; and equilibrium pressure from 2.34 to 5.67 mbar. (**b**) Blank spectrum of the zeolite sample in the 2200 to 3800 cm^−1^ (black line) and after dosing with N_2_ (grey line). The inset shows a magnification of the O–H stretching region. (**c**) van’t Hoff plot for N_2_ adsorbed in Na-Y, data obtained from the IR absorption band at 2336 cm^−1^.

Figure 2a shows FT-IR spectra recorded, over a range of temperature, for N_2_ (fixed dose) adsorbed in Na-Y. A single IR absorption band is seen, which peaks at 2336 cm^−1^. According to abundant literature reports [24,25,26,27], this band is assigned to the N–N stretching mode of the N_2_ molecule interacting end-on with a Na^+^ cation (situated on the internal wall of the zeolite supercage). Such an interaction leads to polarization of the adsorbed molecule and brings about activation in the IR of the N–N stretching vibration, which is only Raman active in the gas phase; at a frequency of 2330 cm^−1^ [25]. Concomitantly, a small hypsochromic shift was expected [28,29], which resulted to be of 6 cm^−1^ in the present case.

For further information, Figure 2b shows a full IR spectrum covering the region of 2200 to 3800 cm^−1^ both, before (black line) and after dosing with nitrogen (grey line). In the N–N stretching region, no other IR absorption band is seen aside from that one at 2336 cm^−1^ already discussed above. In the O–H stretching region two faint bands can be seen, at 3746 and 3698 cm^−1^. The former one is typical of silanols, while the latter is likely to arise from a trace of extra-framework aluminium giving rise to Lewis acid sites (LAS). None of these IR absorption bands seems to be significantly affected by nitrogen, at the temperature and pressure range at which VTIR spectra were recorded. Note also that no IR absorption band is present in the range of 3650–3550 cm^−1^, where framework Si(OH)Al groups (Brønsted acid sites) would be expected to show up [26] in the case of incomplete cation exchange (of the parent ammonic form of the zeolite). Very similar features in the O–H stretching region were also shown by the zeolite samples Ca-Y and Sr-Y.

After computer integration of the IR absorption bands shown in Figure 2a, the corresponding van’t Hoff plot, Figure 2c, of the left-hand side of Equation (5) versus 1/*T* was obtained. From this linear plot, the values of Δ*H*^0^ and Δ*S*^0^ ruling the thermodynamics of the gas-solid adsorption process resulted in −19.7 kJ mol^−1^ and −143 J mol^−1^ K^−1^ respectively. Error limits are estimated to be smaller than ±2 kJ mol^−1^ for enthalpy and ±10 J mol^−1^ K^−1^ for entropy.

### 4.2. Dinitrogen Adsorption in Ca-Y and Sr-Y

**Figure 3:**
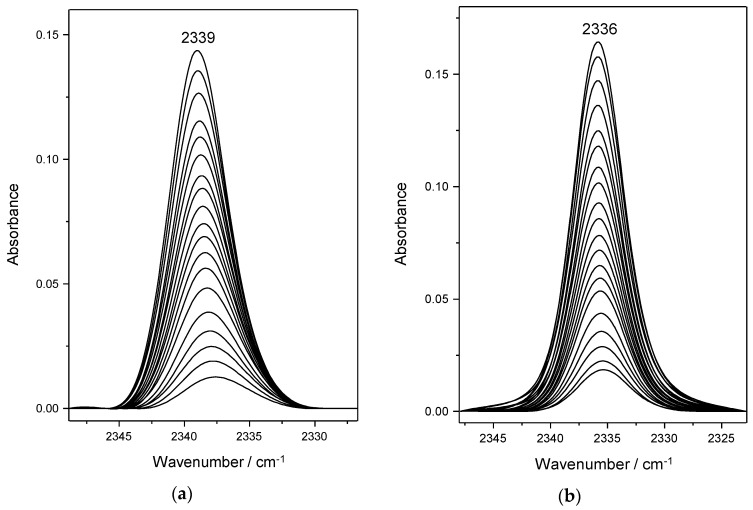
(**a**) Representative variable-temperature IR spectra of N_2_ adsorbed in Ca-Y. From top to bottom temperature increases from 207 to 266 K; and equilibrium pressure from 1.05 to 1.77 mbar. (**b**) Representative variable-temperature IR spectra of N_2_ adsorbed in Sr-Y. From top to bottom temperature increases from 191 to 239 K; and equilibrium pressure from 2.27 to 3.28 mbar.

**Figure 4:**
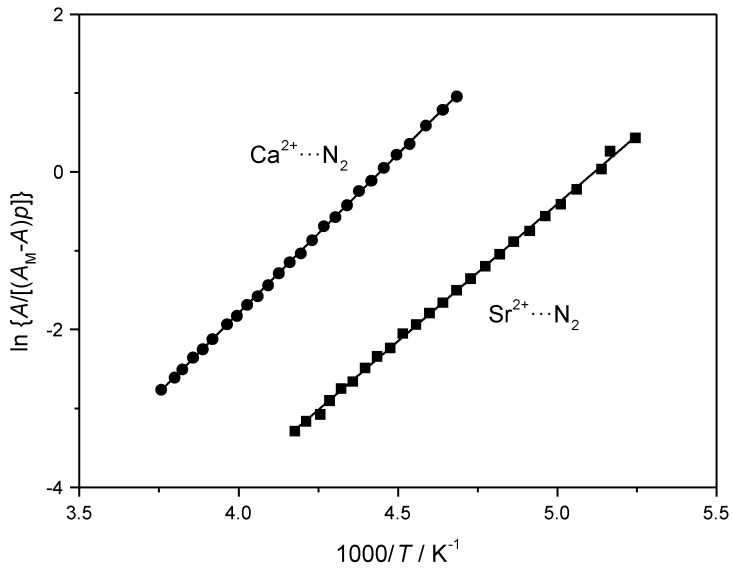
van’t Hoff plots for N_2_ adsorbed in Ca-Y and Sr-Y, data obtained from the IR absorption bands at 2339 (Ca^2+^···N_2_) and 2336 cm^−1^ (Sr^2+^···N_2_), respectively.

Figure 3a depicts representative VTIR spectra, covering the range of 207 to 266 K, obtained for dinitrogen adsorption in the Ca-Y zeolite. The peak wavenumber appears at 2339 cm^−1^, slightly higher than that found for the case of the N_2_/Na-Y system, as expected on account of the larger positive electric charge of the cation. The corresponding plot of the left-hand side of Equation (5) versus the reciprocal of the temperature, for the whole series of spectra recorded, is shown in Figure 4. From this linear plot, the corresponding values of standard adsorption enthalpy and entropy were found to be −33.5(±2) kJ mol^−1^ and −151(±10) J mol^−1^ K^−1^, respectively.

Representative VTIR spectra (191 to 239 K) obtained for dinitrogen adsorption in Sr-Y are shown in Figure 3b. All of them peak at 2336 cm^−1^, this wavenumber value, slightly smaller than that found for the N_2_/Ca-Y system, reflects the smaller polarizing power (charge/radius ratio) of the Sr^2+^ ion as compared to that of Ca^2+^. The van’t Hoff plot for the N_2_/Sr-Y system is shown in Figure 4. Corresponding values of Δ*H*^0^ and Δ*S*^0^ are −29(±2) kJ mol^−1^ and −150(±10) J mo^−1^ K^−1^, respectively.

**Figure 5:**
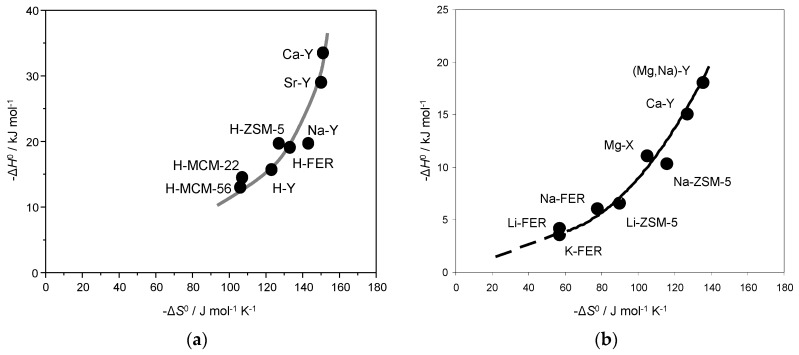
Standard adsorption enthalpy versus entropy for nitrogen (**a**) and hydrogen (**b**) adsorption in several zeolites.

The set of Δ*H*^0^ and Δ*S*^0^ values reported above for N_2_ adsorption in alkaline zeolites is summarized in Table 1, which also compiles corresponding results previously reported [30,31,32] for dinitrogen adsorption in some protonic zeolites. The complete set of results is plotted in Figure 5a, which clearly shows a non-linear enthalpy-entropy correlation. It is relevant to add that a similar correlation between Δ*H*^0^ and Δ*S*^0^ was reported some time ago [17] for the case of dihydrogen adsorption in alkaline zeolites, as shown in Figure 5b. In both cases it turns out that (referring to absolute values) the relative range at which entropy changes gradually slows down as enthalpy increases more and more, thus giving rise to the concave curves seen in Figure 5. Such a non-linear enthalpy-entropy correlation can be rationalized by considering that, in principle, there is no definite limit for Δ*H*^0^ (which increases with increasing interaction energy between the adsorbed molecules and the adsorption site), while Δ*S*^0^ does have an inherent limit, given by complete loss of motion freedom (a limit that is not expected to be attained in physisorption).

In this context, it is also relevant to add that Hercigonja et al. [33] have studied (by adsorption calorimetry) the enthalpy-entropy compensation effect for *n*-hexane adsorption in several ion exchanged ZSM-5 zeolites, finding linear Δ*H*^0^ versus Δ*S*^0^ plots in all cases. By contrast, they found no linear compensation effect for the adsorption of the same gas in faujasite-type zeolites. These findings, taken together with those reported herein for dinitrogen (and dihydrogen) adsorption, suggest that valuable new insights could be expected from further research concerning adsorption enthalpy-entropy correlation effects, which are highly relevant for both gas separation and gas delivery.

## Figures and Tables

**Table 1:** Thermodynamic data for nitrogen adsorbed on several zeolites. Error limits for Δ*H*^0^ and Δ*S*^0^ are ±2 kJ mol^−1^ and ±10 J mol^−1^ K^−1^, respectively.

Zeolite	−Δ*H*^0^ (kJ mol^−1^)	−Δ*S*^0 *b*^ (J mol^−1^ K^−1^)	Ref
H-MCM-56	13	106	[32]
H-MCM-22	14.5	107	[32]
H-Y	15.7	123	[30]
H-FER	19.1	133	[30,31]
H-ZSM-5	19.7	127	[30]
Na-Y	19.7	143	This work
Sr-Y	29	150	This work
Ca-Y	33.5	151	This work

***^b^*** Referred to a standard state at 1 mbar. Within the perfect gas approximation, Δ*S*^0^ changes by an amount of +57 J mol^−1^ K^−1^ when referred to a standard state at 1 bar.
